# Exploration on the mechanism of crystal morphology transformation in mordenite†

**DOI:** 10.1039/d5ra00666j

**Published:** 2025-03-20

**Authors:** Zijian Wang, Ming Ke, Zhaozheng Song

**Affiliations:** a College of Science, China University of Petroleum Beijing 102249 China song@cup.edu.cn

## Abstract

Mordenite is extensively utilized in the domains of adsorption and catalysis owing to its excellent selectivity, stability, and renewability. In this work, the effects of crystal seeds, the ratio of SiO_2_/Al_2_O_3_, crystallization temperature, silicon source, and structure directing agents on the morphologies of mordenite are investigated by utilizing hexamethyleneimine (HMI) as the OSDA. Results indicate that the transformation of rod-like and flake-like mordenites is mainly related to the increasing ratio of SiO_2_/Al_2_O_3_, and the formation of flake-like morphology is related to various conditions. On a macro level, the rod-like and flake-like zeolites are formed by transforming amorphous precursors into small crystals and growing them epitaxially. On a micro level, HMIH^+^ progressively displaces Na^+^ to balance the framework charge with the increasing ratio of SiO_2_/Al_2_O_3_. DFT calculation and nuclear magnetic resonance results confirm the specific guidance of HMI on the T_2_ and T_4_ sites of the rod-like and flake-like mordenites. The conclusion can serve as a reference for regulating the morphology and active sites of mordenite.

## Abstract 2

The transformation of rod-like and flake-like mordenites is mainly related to the increasing ratio of SiO_2_/Al_2_O_3_, and HMI guides the T_2_ and T_4_ sites of the rod-like and flake-like mordenites specifically.

## Graphical abstract

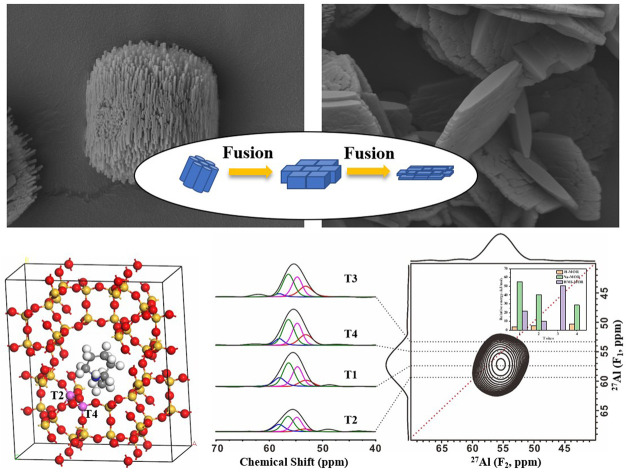

## 1 Introduction

As a type of zeolite studied earlier, mordenite is obtained naturally^1^ or through artificial synthesis.^2^ The crystal belongs to the orthorhombic crystal system with the *Cmcm* space group. The lattice constants are *a* = 18.094 Å, *b* = 20.516 Å, and *c* = 7.524 Å.^3^ Mordenite has a two-dimensional pore structure. Specifically, it consists of a 12-membered ring (12-MR) channel with a pore diameter of 6.5 Å × 7.0 Å and an 8-membered ring (8-MR) channel with a pore diameter of 2.6 Å × 5.7 Å,^4^ which runs parallel to the 12-MR channel. The 12-MR and 8-MR channels are interconnected *via* an additional slim and winding 8-MR side pocket, which has a dimension of 3.4 Å × 4.8 Å. The 8-MR side pocket plays an important role in catalytic reactions such as the carbonylation of dimethyl ether,^5^ while the 12-MR channel is involved in larger molecule reactions such as toluene disproportionation and alkylation transfer.^6^

The morphology of mordenite affects its specific catalytic performance. Ma *et al.*^7^ found that flake-like mordenite had a higher dimethyl ether conversion rate compared with traditional zeolite with similar SiO_2_/Al_2_O_3_ ratios, which was attributed to the fact that 12-MR was more conducive to the diffusion of molecules. Li *et al.*^8^ found that the initial activity of mordenite was positively correlated with the ratio of L_*c*_/L_*b*_ and the catalytic rate after 2 hours was directly proportional to the ratio of L_*c*_/L_*b*_ in the dimethyl ether carbonylation reaction. In comparison with Ma *et al.*,^7^ the difference was related to the range of the *c*-axis length, indicating that the length range may lead to different relationships between the catalytic activity and *c*-axis length.

Two-dimensional zeolites are widely investigated in recent years,^9^ such as MFI, MWW, and MOR. The short diffusion distance results in less carbon deposition to enhance the catalytic activity and stability in specific reactions. Compared with post-synthesis,^10^*in situ* hydrothermal synthesis is more accessible and cheaper. Conventional hydrothermal synthesis of zeolites includes the single template method, the double template method, and the control of synthesis conditions. The dual-functional template method often involves the use of multi-headed quaternary ammonium salts^7,11–13^ which affect the crystal thickness, interlayer spacing and ordering, and the double template method exhibits variations in its synergistic mechanisms. For example, *N*,*N*,*N*-trimethyl-1,1-adamantyl ammonium hydroxide (TMAda^+^) and 1,2-hexanediol are considered to guide the formation of the 12-MR channel and 8-MR side pocket of mordenite, respectively,^14^ while HMI and TEAOH are believed to promote the crystallization of MWW topology and hinder Si–O–Al bridge formation in interlayers, respectively.^15^ The variation in template content may also have distinct effects in the process of crystallisation.^16^ Compared with other methods, the low-cost single template method shows greater potential for controlling the morphologies and acid distribution of zeolites.

Apart from the synthesis method without utilizing organic structure-directing agents (OSDAs),^17^ the synthesis of mordenite often involves tetraethylammonium (TEA^+^)^18^ as the single template. This often leads to the formation of a spherical morphology growing in three-dimensions or a rod-like morphology growing along the *c*-axis. Hexamethyleneimine (HMI) displays high flexibility due to its considerable number of methylene groups, which can effectively participate in synthesizing a wide range of zeolites such as MCM-22,^19^ MCM-49,^20^ MOR,^21^ and ZSM-5.^22^ Organic amines and alkaline cations play an important role in determining the morphologies and acidic sites of zeolites.^23^ Herein, the influencing factors of the mordenite morphology are investigated, specifically focusing on the transformation of rod-like and flake-like structures. The growth of these is analyzed on both macroscopic and microscopic levels. This work offers a theoretical foundation for utilizing HMI as the OSDA to synthesize mordenite.

## 2 Experimental section

### 2.1 Materials

Mordenite samples were synthesized utilizing the following chemicals without any purification. Acidic silica sol (SiO_2_, 30 wt%, Dezhou Jinghuo Technology Glass Company), silicic acid (SiO_2_, 77 wt%, Shanghai Aladdin Biochemical Technology Company), fumed silica (SiO_2_, 99 wt%, Jiangsu Tianxing New Materials Company), and tetraethylorthosilicate (SiO_2_, 29 wt%, Shanghai Aladdin Biochemical Technology Company) were used as silicon sources. Sodium metaaluminate (Al_2_O_3_, 45wt%, Tianjin Guangfu Fine Chemical Research Institute), hexamethyleneimine (HMI, 98 wt%, Shanghai Aladdin Biochemical Technology Company Limited), sodium hydroxide (NaOH, 96 wt%, Tianjin Fuchen Chemical Reagent Company), potassium hydroxide (KOH, 99 wt% Shanghai Macklin Biochemical Technology Company), nano-mordenite (Liaoning Raodong New Materials) and ammonium chloride (NH_4_Cl, 99.5 wt% Tianjin Guangfu Fine Chemical Research Institute) were used for the preparation of mordenite.

### 2.2 Mordenite preparation

The typical synthesis of mordenite was performed as follows. Sodium hydroxide and sodium aluminates were added in deionized water with stirring for 30 minutes. Hexamethyleneimine and silicon sources were added to the above solution with stirring for 30 minutes. Tetraethylorthosilicate had to be pre-hydrolyzed, which was different from other silicon sources. Specifically, tetraethylorthosilicate was added gradually to the mixture of hexamethylene and deionized water, and the mixture was stirred for 30 minutes. The solution of sodium hydroxide and sodium metaaluminate was added slowly into the one mentioned above. The crystal seed was finally added to the synthetic gel with the final molar composition of SiO_2_ : 0.05–0.5Na_2_O : 0.02–0.1Al_2_O_3_ : 0.3HMI : 25H_2_O : 0.03seed (mass). The gel was stirred at 50 °C for 24 h and transferred to Teflon-lined autoclaves under static crystallization. The Na(K)-type products were obtained by filtering, washing to neutral, drying and calcination in air at 550 °C for 6 h. H-type samples were obtained by ion exchange in 1 M NH_4_Cl solution at 90 °C for 6 h, filtering, washing to neutral, drying and calcination in air at 550 °C for 6 h.

### 2.3 Characterization

X-ray diffraction (XRD) patterns were obtained utilizing a Bruker D8 Advance X-ray diffractometer with Cu-Kα radiation (*λ* = 1.5406 Å) operating at 40 kV and 40 mA. The sample morphologies were measured on a Gemini SEM 300 scanning electron microscope (SEM). Transmission electron microscopy (TEM) images were obtained utilizing an FEI Tecnai F20 instrument, operating at an accelerating voltage of 200 kV. Elemental analyses (Si, Al and Na) for the samples were performed by using an inductively coupled plasma-atomic emission spectrometer (ICP-OES, Agilent 5110) after dissolving the products in aqua regia solution. Fourier transform infrared (FT-IR) spectra were recorded on a Thermo instrument utilizing the KBr disc technique. The TGA measurements were carried out under an air atmosphere on a Discovery TGA 55 instrument. ^27^Al MAS NMR and 2D ^27^Al MQ MAS NMR experiments were performed at 18.8 T magnetic field on an Avance Neo 600 spectrometer with the corresponding Larmor frequency of 156.4 MHz.

### 2.4 Computational models and methods

The periodic model of mordenite was obtained from the IZA structure database.^3^ To consider both the accuracy and efficiency, the computation structure used a single unit cell model consisting of 96 O atoms and 48 Si atoms. Mordenite possessed 4 nonequivalent T sites and 10 O sites. The negative charges induced by the incorporation of AlO_2_^−^ were compensated by Na^+^ or HMIH^+^. The energies of Si atoms substituted by the Al atoms of the Na-MOR or HMI-MOR compound were calculated to compare the directing effect of different SDAs on Al distribution in the MOR framework.^24,25^ The substitution energy was calculated as depicted in formulas (1) and (2).^26^

1 Si-MOR + Al(OH)_3_ + SDA^+^OH^−^ = SDA-MOR + Si(OH)_4_

2 *E*_sub_ = *E*_SDA-MOR_ + *E*_Si(OH)_4__ − *E*_Si-MOR_ − *E*_Al(OH)_3__ − *E*_SDA^+^OH^−^_

The periodic density functional theory (DFT) as well as Monte Carlo (GC-MC) simulations were carried out by Materials Studio software. The generalized gradient approximation (GGA) and Perdew–Burke–Ernzerhof (PBE) exchange–correlation functional were used, and the TS method was utilized for DFT-D correction,^27^ with the D-polarization function augmented double numerical atomic orbitals (DNP) as the basis set.^28^ The calculation criteria for the self-consistent field (SCF) with the effective nuclear potential (ECP) were 10^−6^ Ha. The convergence criteria of total energy, force and displacement were 1 × 10^−5^ Ha, 2 × 10^−3^ Ha Å^−1^ and 5 × 10^−3^ Ha Å^−1^, respectively. The Monte Carlo simulation process adopted the Metropolis method and Dreiding force field^29,30^ and the charges were applied utilizing the DFT simulation results. The long-range electrostatic interaction was calculated with the Ewald summation method, and the Lennard-Jones interaction with a cut-off radius of 18.5 Å.

## 3 Results and discussion

### 3.1 Effect of seeds

Seed-assisted synthesis (SAS) is a common method in the field of porous material synthesis, which is used not only in zeolites but also in MOFs and COFs.^31–33^ The role of crystal seeds in the growth of zeolites is complex. At present, it is widely accepted that there exist two mechanisms, namely dissolution and retention,^34^ which exert an influence on the physical and chemical characteristics of the synthesized zeolites. Besides, the inorganic alkali involves the influence of alkaline cations and the process of mineralization facilitated by OH^−^ ions.^35^

**Fig. 1:**
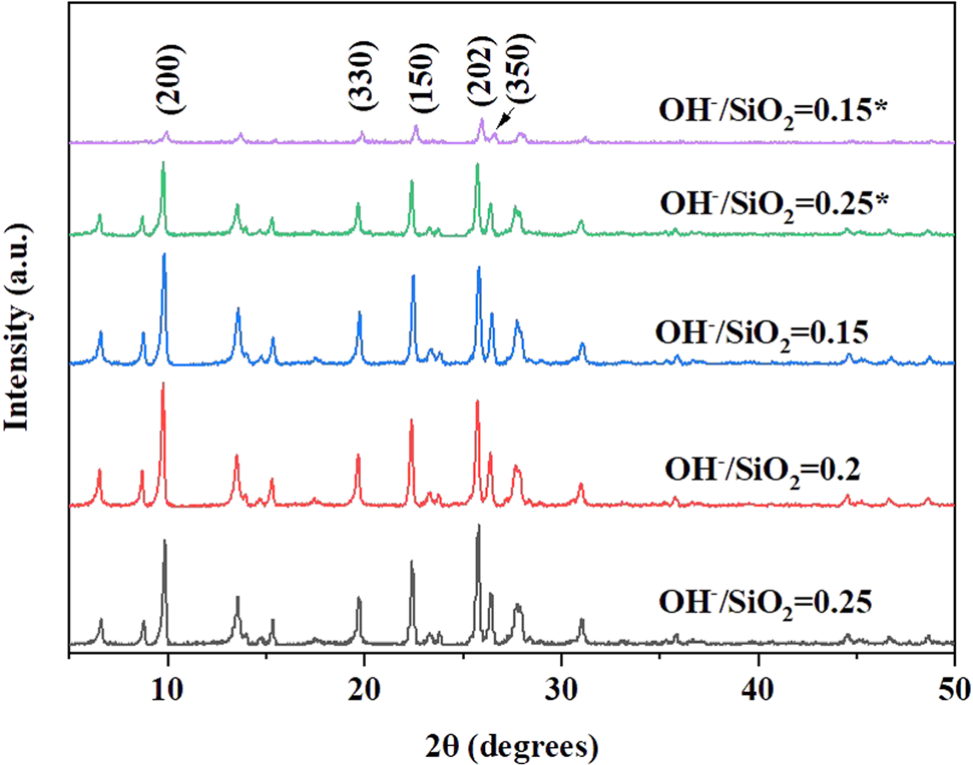
XRD patterns of H-type samples with different ratios of OH^−^/SiO_2_ (*without seeds).

**Fig. 2:**
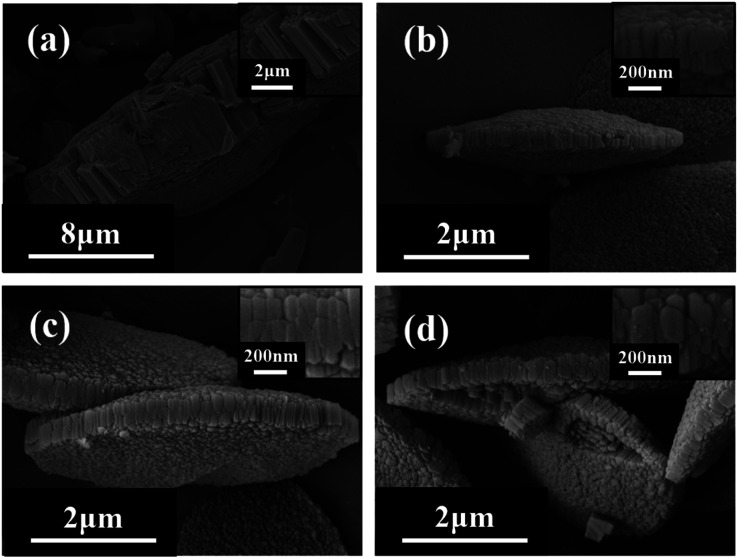
SEM images of H-type samples with different ratios of OH^−^/SiO_2_ ((a) 0.25*; (b) 0.15; (c) 0.2; (d) 0.25) (*without seeds).

Under the conditions of SiO_2_/Al_2_O_3_ ratio of 21 and crystallization temperature of 150 °C, XRD patterns (Fig. 1) show that the faint mordenite diffraction peaks of the gel with OH^−^/SiO_2_ = 0.15 will only emerge in 7 days. When the OH^−^/SiO_2_ of the gel increases to 0.25, mordenite grows completely in 4 days. And it is because higher alkalinity can dissolve amorphous SiO_2_ and Al_2_O_3_ into free state Si–OH and Al–OH units in a shorter time, which is favorable for Si–OH and Al–OH to recombine to form SiO_4_ and AlO_4_ tetrahedra of the mordenite framework.^36,37^ The gel with OH^−^/SiO_2_ = 0.15–0.25 can be used to form mordenite in 4 days with the assistance of crystal seeds, which suggests that the crystal seed is able to expand the range of OH^−^/SiO_2_ and influence the size of zeolite (Fig. 2). In this work, the dissolution mechanism of crystal seeds might be the primary factor according to the relatively low seed content in zeolite growth mixtures^34^ and the SEM image of crystal seeds (Fig. S2†) which is different from the target zeolite in morphology and size. The dissolved species may be the oligomers that lack a high degree of local order and impact on nucleation,^38–40^ changing the synthesis conditions and the crystal size.

### 3.2 Effect of SiO_2_/Al_2_O_3_

**Fig. 3:**
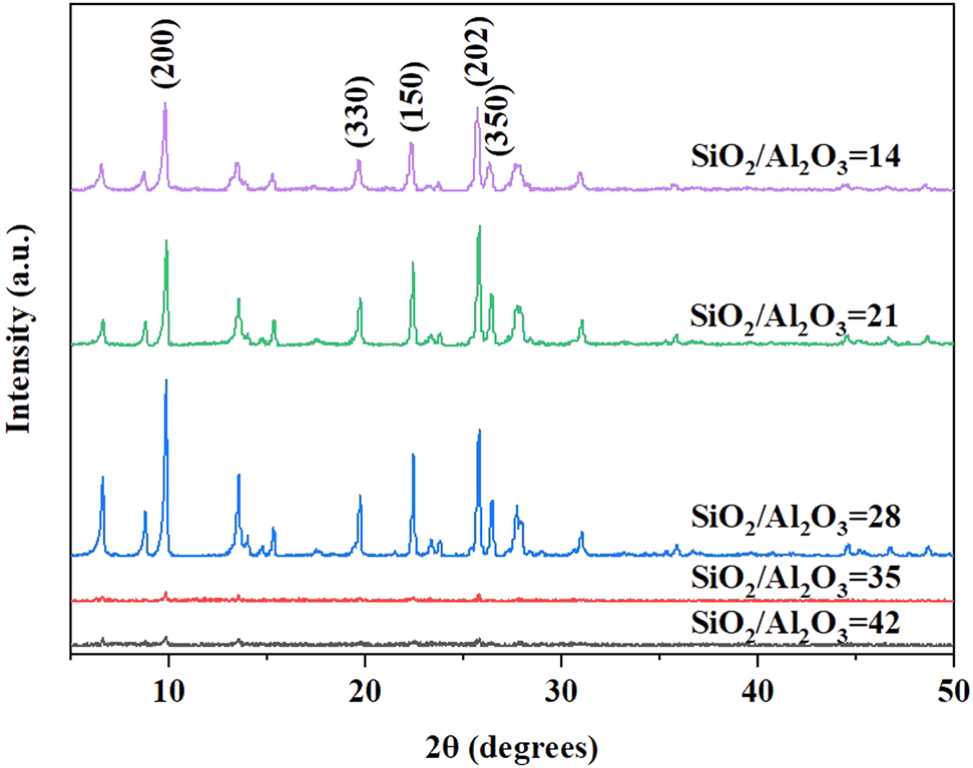
XRD patterns of H-type samples with different ratios of SiO_2_/Al_2_O_3_ (150 °C).

**Fig. 4:**
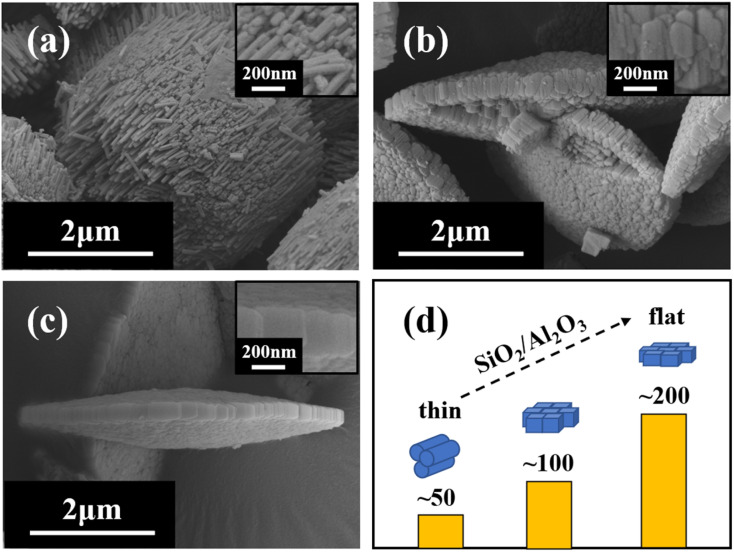
SEM images of H-type samples with different ratios of SiO_2_/Al_2_O_3_ ((a) 14; (b) 21; (c) 28) and process of layer width transformation (d).

Samples with different ratios of SiO_2_/Al_2_O_3_ under the gel with OH^−^/SiO_2_ = 0.25 and 3 wt% crystal seed as well as the crystallization temperature of 150 °C conditions are chosen for further investigation. XRD patterns (Fig. 3) and SEM images (Fig. 4) show that the rod-like mordenite can be synthesized when the ratio of SiO_2_/Al_2_O_3_ ranges from 14 to 28. The small rod-like crystal undergoes a progressive transformation from thin to flat as the ratio of SiO_2_/Al_2_O_3_ increases and the entire crystal finally becomes a flake-like morphology. Hincapie *et al.*^41^ found that mordenite with small crystal size could be obtained under low ratio of SiO_2_/Al_2_O_3_, which was explained by means of Lowenstein's rule. The different connection mode of silicon species from that of aluminum species may also be responsible for the different crystal growth modes and result in the different morphologies under the conditions of various ratios of SiO_2_/Al_2_O_3_.

**Fig. 5:**
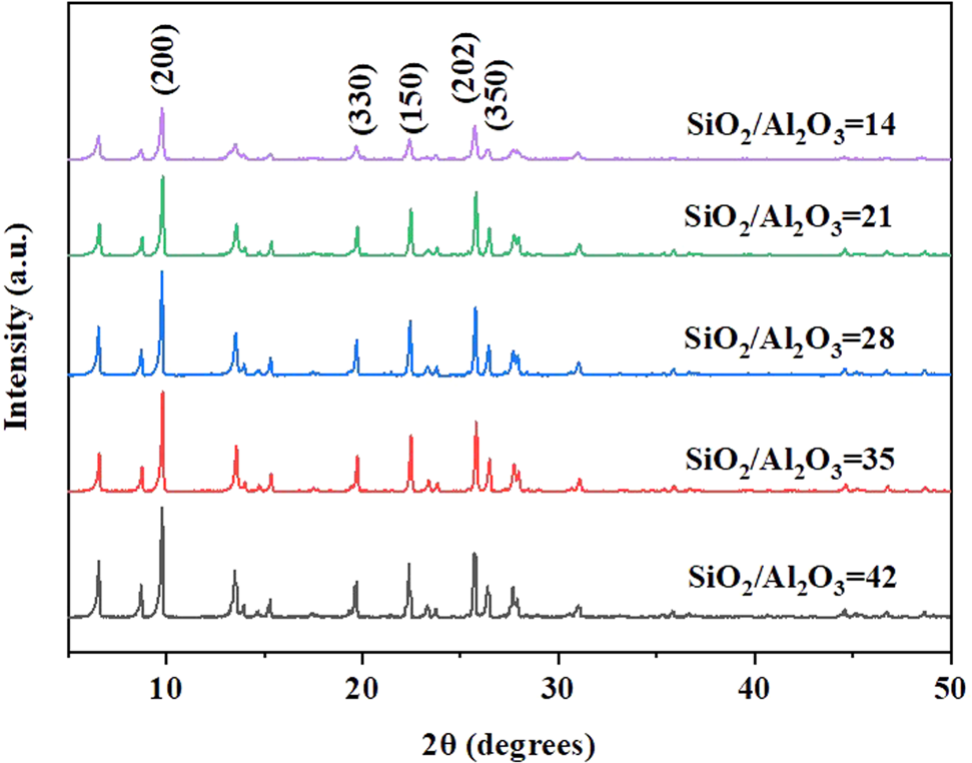
XRD patterns of H-type samples with different ratios of SiO_2_/Al_2_O_3_ (170 °C).

**Fig. 6:**
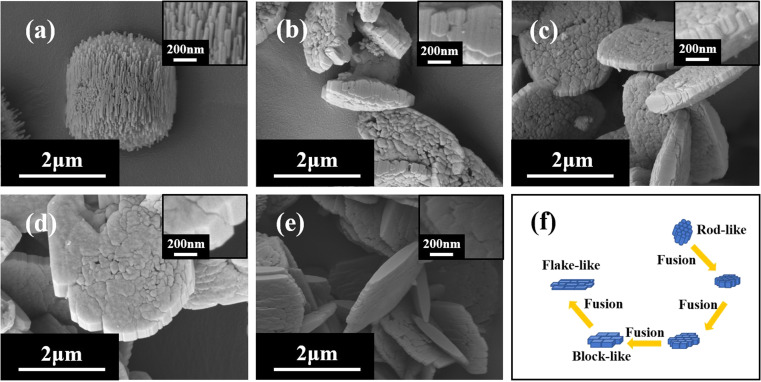
SEM images of H-MOR with different ratios of SiO_2_/Al_2_O_3_ ((a) 14; (b) 21; (c) 28; (d) 35; (e) 42) and transformation process (f).

XRD patterns (Fig. 5) show that the mordenite can be synthesized in 2 days under the crystallization temperature of 170 °C and the ratio of SiO_2_/Al_2_O_3_ can be extended to 42. Compared with the low temperature, distinct rod-like morphologies are only formed when the ratio of SiO_2_/Al_2_O_3_ ranges from 14 to 21 (Fig. 6). Different morphologies might result from the slower rate of crystallisation at lower temperature which promotes the development of crystal facets with high density (*c*-axis).^42^ With the increasing ratio of SiO_2_/Al_2_O_3_, the crystal undergoes a transition from a rod-like morphology to a block-like morphology, and ultimately converts into a flake-like morphology.

**Fig. 7:**
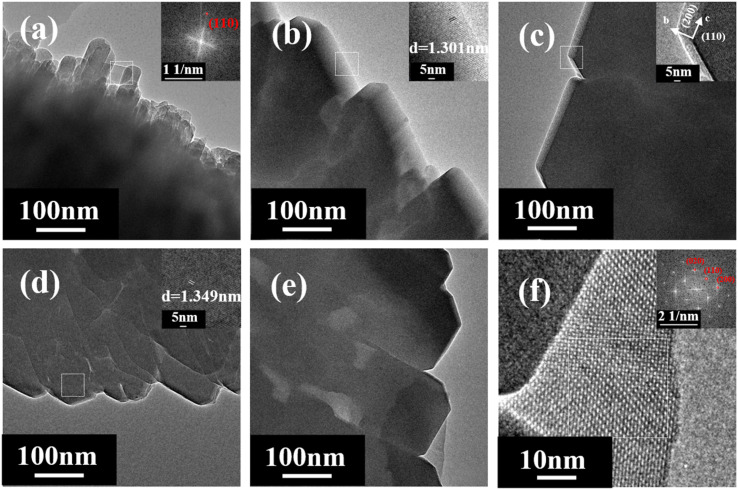
TEM images of H-type samples with different SiO_2_/Al_2_O_3_ ratios ((a) 14; (b) 21; (c) 28; (d) 35; (e and f) 42).

TEM/HRTEM images in Fig. 7 show the crystal morphology and internal layer-structure of each sample. TEM images (Fig. 7a–e) also indicate that as the ratio of SiO_2_/Al_2_O_3_ increases, the morphology of mordenite transforms from a rod-like to a flake-like morphology. According to the analysis of electron diffraction patterns and interplanar spacing, it can be concluded that the rod-like sample (SiO_2_/Al_2_O_3_ = 14) is parallel to the *c*-axis direction. However, the *c*-axis direction of other samples is consistent with the layer structure. Liu *et al.*^43^ found that K^+^ could break the hydrogen bonds of water molecules, but the interaction was not sufficient to form hydrated cations, which affected the dissolution and mass transfer during the crystallization process. As a result, the growth rate of zeolite along the [001] crystal facet was lower than that along the [110] crystal facet, forming a wheel-like crystal with a *c*-axis length of 200–300 nm. In this work, the crystals synthesized utilizing Na^+^ have a higher degree of fusion and form different crystal orientations, which indicates that alkaline cations may play a role in the growth of crystal facets.

### 3.3 Effect of silicon source

**Fig. 8:**
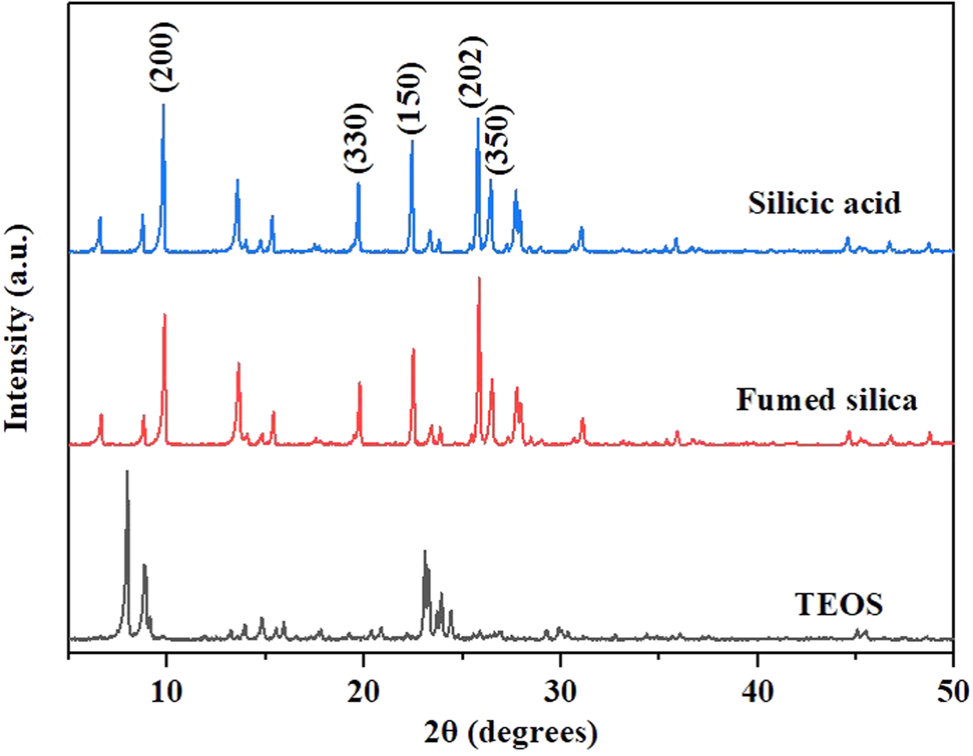
XRD patterns of H-type samples from different silicon sources.

**Fig. 9:**
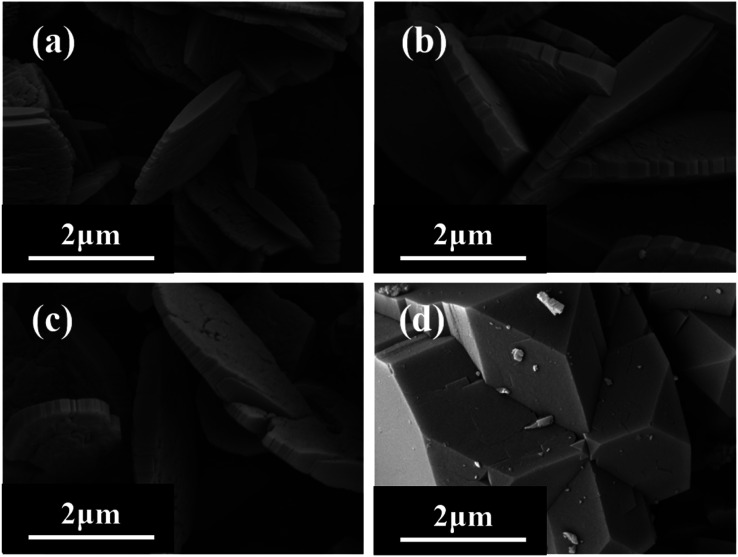
SEM images of H-type samples from different silicon sources ((a) acidic silica sol; (b) silicic acid; (c) fumed silica; (d) TEOS).

Silicic acid, fumed silica, and tetraethylorthosilicate (TEOS) are chosen as comparative silicon sources. XRD patterns (Fig. 8) show that mordenite can be synthesized by utilizing silicic acid and fumed silica as silicon sources and the samples exhibit a block-like morphology (Fig. 9). The different morphologies might result from the difference in the release of silicon species into liquid medium which changes the growth rates of crystal facets.^44^ The crystal synthesized by utilizing TEOS as the silicon source is MFI zeolite, which has a morphology resembling agglomerated prisms. In fact, the ethanol generated by TEOS hydrolysis might reduce the concentration of silicon and aluminum species in aqueous solutions^45^ and affect the type of crystal. Zhang *et al.*^46^ indicated that the formation of MFI zeolite typically necessitated the usage of active seeds with silanol groups on the surface when HMI was used as the OSDA. Additionally, it requires more serious conditions for the composition of synthetic gels.^22^ In this work, mordenite as the heterologous seed assisting the formation of MFI zeolite has been discovered. Li *et al.*^47^ found that the synthesis of MFI zeolite assisted by crystal seeds should have at least one of two conditions. On one hand, the framework density of the crystal seed was not higher than that of the target structure. On the other hand, there were common CBUs/SBUs between the crystal seed and the target zeolite. Mordenite not only has the lower framework density (17.0 T/1000 Å^3^) than MFI zeolite (18.4 T/1000 Å^3^), but also has the same CBUs(mor) as MFI zeolite, which helps to overcome the kinetic hurdles during transformations, especially for assisting the nucleation.^17^

### 3.4 Effect of SDA

**Fig. 10:**
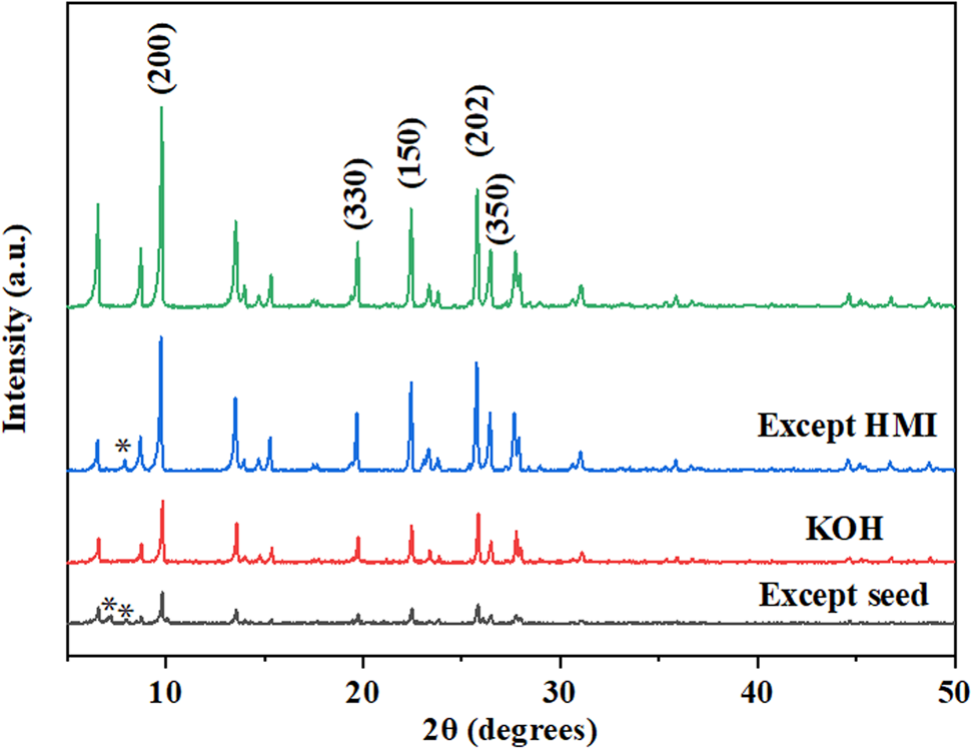
XRD patterns of H-type samples from different SDAs (*other phases).

**Fig. 11:**
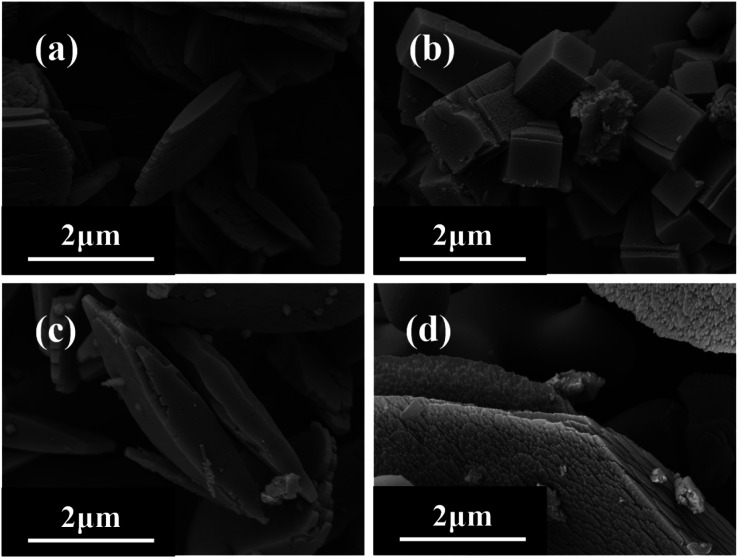
SEM images of H-type samples from different SDAs ((a) MOR-42; (b) KOH; (c) except HMI; (d) except seed).

The crystal synthesis is influenced by the structure directing agents (SDA) such as alkaline cation,^23^ crystal seed,^17,43^ and organic structure directing agent (OSDA). In this work, the main factors are considered, which are replacing NaOH with KOH as the alkali source and excluding the crystal seed or OSDA. XRD patterns (Fig. 10) and SEM images (Fig. 11) show that the crystal seed not only promotes the growth of zeolite, but also diminishes the generation of heterocrystals. When KOH is used as the alkali source, the crystal cannot grow completely in 2 days and the morphology is significantly different. Na^+^ not only contributes to the growth of zeolite, but also influences its morphology. Vuono *et al.*^48^ found that Na^+^ had an impact on the growth of MCM-22(P) zeolite, and attributed it to the unique structural guidance of Na^+^ which contributed to the incorporation of Al into the crystal framework. In addition, the pure sample cannot be synthesized and has a lower relative crystallinity (79.91%) in the absence of HMI. Liu *et al.*^49^ discovered that employing HMI as the OSDA enhanced the relative crystallinity of mordenite and attributed the formation of the flake-like structure to the synergetic effect of TEAOH and HMI. However, in this work, the ratio of SiO_2_/Al_2_O_3_ and Na^+^ play a role in the formation of the flake-like structure instead of HMI. Organic structure directing agents (OSDAs) not only have template and structure-directing effects, but also can fill the channels of zeolites, which may diminish the generation of heterocrystals. Therefore, it is clear that the synthesis of flake-like mordenite involves the important participation of crystal seeds, Na^+^, and HMI.

### 3.5 Exploration of the synthesis mechanism

#### 3.5.1 Exploration of apparent growth

Samples which have the ratio of SiO_2_/Al_2_O_3_ = 14 and 42 are chosen for further investigation into the growth mechanism of rod-like and flake-like mordenites. The growth curve of mordenite is calculated based on relative crystallinity. To be specific, the calculation method for determining the relative crystallinity of mordenite involves measuring the integrated area of eight specific diffraction peaks at angles of 2*θ* = 9.7°, 13.5°, 19.7°, 22.4°, 25.7°, 26.4°, 27.6°, and 27.8°.^50^

**Fig. 12:**
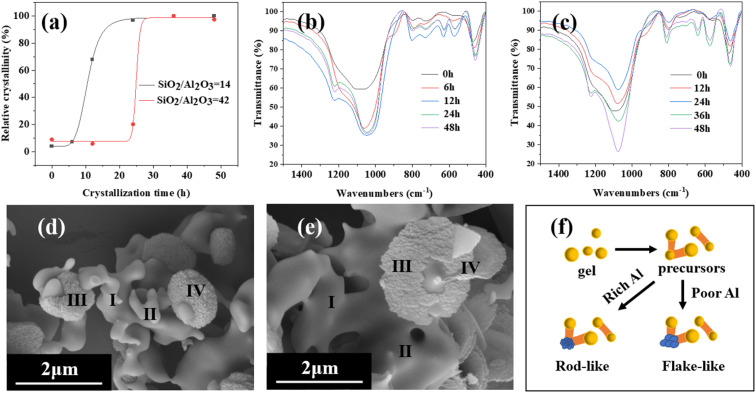
(a) Crystallization curves of samples (SiO_2_/Al_2_O_3_ = 14 and 42); (b and c) FT-IR spectra of samples (SiO_2_/Al_2_O_3_ = 14 and 42); (d and e) SEM images of samples (SiO_2_/Al_2_O_3_ = 14 and 42); (f) comparison of growth processes.

The XRD patterns (Fig. 12a) show that the crystallization curves of the two samples exhibit a typical sigmoidal shape and the rod-like mordenite grows more rapidly than the flake-like mordenite. The lower ratio of SiO_2_/Al_2_O_3_ might be advantageous for the formation of 4-MR structures^51^ and nucleation. It undergoes a transition from amorphous precursors, resembling worm-like particles, to a structured crystal at 6 and 12 hours, respectively. Then, the cake-like and flake-like tiny crystals grow epitaxially to form complete crystals. The microstructure of precursors is not fully understood.^52^ And the research on the nonclassical growth mechanism shows that there may exist two types of attachment manners between precursors and crystals, namely, side-by-side coalescing and flat-by-flat stacking.^53^

FT-IR spectra show that the synthetic gel of the two samples lacks distinct absorption peaks in the vicinity band of 580 cm^−1^ and 560 cm^−1^. On one hand, there might not be any formation associated with the secondary building units. On the other hand, the secondary building units are not externally connected.^54^ As the duration of crystallisation increases, there is no noticeable shift towards higher frequencies in the vibration band at 1050 cm^−1^, which suggests that the proportion of Si and Al in the solid solution remains relatively unchanged.^55^ The EDS analysis of rod-like mordenite reveals that the ratios of SiO_2_/Al_2_O_3_ from amorphous phases are 11.96 (I) and 11.84 (II), respectively, and the ratios of SiO_2_/Al_2_O_3_ from crystal phases are 11.55 (III) and 12.27 (IV), respectively. And the result of flake-like mordenite indicates that the ratios of SiO_2_/Al_2_O_3_ from amorphous phases are 25.14 (I) and 24.46 (II), respectively, and the ratios of SiO_2_/Al_2_O_3_ from crystal phases are 24.56 (III) and 23.03 (IV), respectively. However, Zhang *et al.*^42^ found that the ratio of Si/Al from crystal and bulk gel had a relationship with the crystallization time, which was due to the difference in solubility of silicon and aluminum species and the heterogeneity of the starting synthesis gel. As a result, during the growth of the two types of mordenite, silicon and aluminium may similarly transform from the amorphous phase to the crystal phase from the perspective of the solid-phase transition mechanism in this work.

#### 3.5.2 Exploration of microscopic growth

The apparent growth exploration reveals that rod-like zeolites and flake-like zeolites are formed by epitaxial growth with relatively uniform migration rates of Si and Al, respectively. The guiding effect of the SDA on specific Al sites requires further analysis through DFT calculation and nuclear magnetic resonance. Na^+^, crystal seed, and HMI are discovered as structure-directing agents in the synthesis of mordenite with a flake-like structure. The alteration of the crystal morphology is believed to be influenced not only by the crystal seed and crystallisation temperature, but also by the interplay between the structural orientations of Na^+^ and HMI, which can be both synergistic and competing. Iorio *et al.*^23^ believed that the TMAda^+^ and alkaline cation balanced the negative charges in synthetic zeolites. In this work, HMI can be estimated by calculating the decrease in weight between 433 K and 923 K in an air atmosphere.^56^

Table S1† shows that the ratios of SiO_2_/Al_2_O_3_ from the synthetic gel are positively correlated with those of the zeolite. The higher alkalinity facilitates the dissolution of Al_2_O_3_ in the gel, resulting in a higher Al content in the reaction system.^57^ Meanwhile, the concentration of Na/Al (mol) falls gradually, whereas the concentration of HMI/Al (mol) increases gradually. It can be inferred that as the ratio of SiO_2_/Al_2_O_3_ increases during the formation of mordenite, HMIH^+^ gradually replaces Na^+^ to balance the framework charge. The image (Fig. 11c) shows that the presence of HMI does not primarily contribute to the creation of the flake-like structure. However, it does play a role in stabilizing the crystal and regulating the balanced framework charge.

**Fig. 13:**
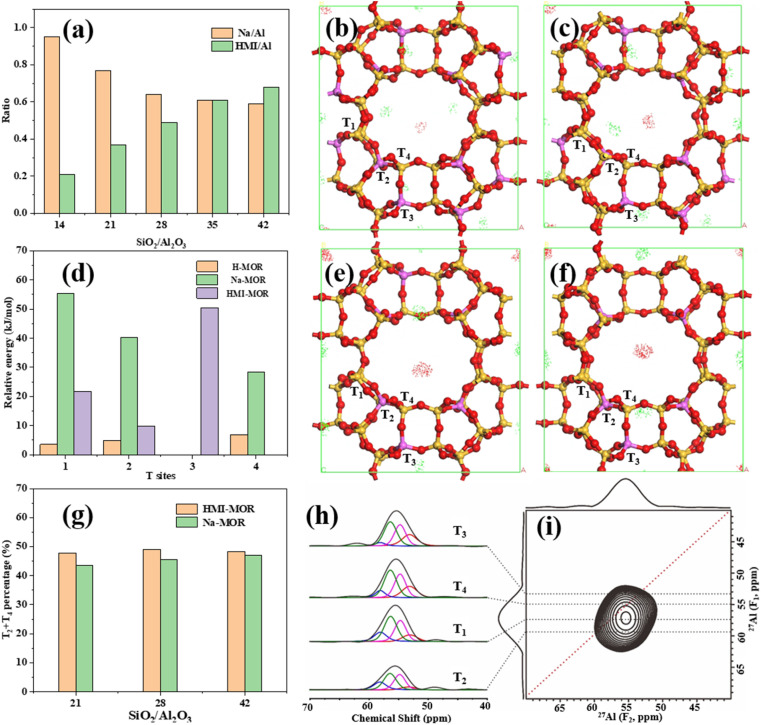
(a) Ratio of Na/Al and HMI/Al from different samples; (b, c, e and f) GCMC simulations of different samples; (d) relative energies of different T sites; (g) T_2_ + T_4_ percentage of HMI-MOR and Na-MOR from different samples; (h and i) 2D ^27^Al MAS NMR spectra and analysis of different T sites.

Grand Canonical Monte Carlo (GC-MC) simulations (Fig. 13b, c, e and f) are conducted to determine the possible position of Na^+^ and HMIH^+^ in the framework. The ratios of SiO_2_/Al_2_O_3_ from models are 6.7, 8.7, 14, and 17.2. The substitution of Al occurs randomly and does not break Löwenstein's rule. The ratios of Na^+^/HMIH^+^ are based on the analysis mentioned above. It is apparent that HMIH^+^ is primarily adsorbed in the 12-MR main channel, whereas Na^+^ can be adsorbed in the 12-MR channel, 8-MR channel and side pocket among the models. Chibani *et al.*^58^ indicated that the most stable position for Na^+^ was in the 8-MR channel because the framework had higher energies when it was in the main channel and side pocket. The impact of Na^+^ and HMIH^+^ on the positioning of Al in the mordenite framework was analyzed. The substitution energy of H-MOR was computed to compare with Na-MOR and HMI-MOR. The Brønsted centres are chosen as T_1_O_7_, T_2_O_5_, T_3_O_1_, and T_4_O_2_.^59^ And the initial positions for HMIH^+^ and Na^+^ are determined based on the GCMC simulation result and ref. 58. As shown in Fig. 13d, the most stable site for H-MOR is T_3_O_1_ and other non-equivalent sites have similar substitution energy, which is consistent with Guo *et al.*^59^ Nevertheless, it is difficult for H^+^ to directly balance the charge of the framework to form H-MOR in the conventional hydrothermal synthesis. In this work, Na^+^ and HMIH^+^ balance the as-synthesized samples' charge together according to the ICP-OES and TG analysis. The most stable site for Na-MOR is the T_3_ site, whereas the T_1_, T_2_ and T_4_ sites have higher substitution energies of 55.33 kJ mol^−1^, 40.29 kJ mol^−1^, and 28.47 kJ mol^−1^, respectively. Chibani *et al.*^58^ also believed that the T_3_ site was the most stable while the T_1_ site was the most unstable for Na-MOR. When HMIH^+^ balances the framework charge, the T_4_ site becomes the most stable, whereas the T_1_, T_2_, and T_3_ sites have higher substitution energies of 21.80 kJ mol^−1^, 9.87 kJ mol^−1^, and 50.51 kJ mol^−1^, respectively.

Solid-state nuclear magnetic resonance is used to identify and quantitatively analyze different framework aluminum species in the MOR-*x* sample. The MOR-21 sample shows good distinction at a magnetic field strength of 18.8 T (Fig. 13i). According to the F_1_ dimension information, the chemical shifts of different framework aluminum species in the F_2_ dimension are identified as T_3_, T_4_, T_1_ and T_2_.^5^ The DFT simulation result (Fig. 13d) indicates that HMI has lower substitution energies at T_2_ and T_4_ sites, while Na^+^ has lower substitution energies at T_3_ and T_4_ sites. Vilhena *et al.*^60^ also suggested that Na^+^ exhibited a preference for adsorption on T_3_ and T_4_ sites by the DFT simulation. Samples with SiO_2_/Al_2_O_3_ ratios of 21, 28, and 42 are synthesized for comparison without the addition of HMI. The analysis results are shown in Fig. S11 and Table S3.† The nuclear magnetic resonance result confirms that the total amounts of T_2_ + T_4_ sites under the synthesis condition of HMI as the OSDA are higher than those under the synthesis condition of Na^+^ as the SDA. However, the substitution energy of the single site differs from the relative content change of the SDA. On one hand, there are differences in the effects of multiple OSDAs^61^ and complex conditions on the Al sites.^56^ On the other hand, the formation process of the zeolite framework is usually metastable and in some cases not thermodynamically driven.^62^

## 4 Conclusion

The effects of various conditions on the morphologies of mordenite are investigated by utilizing hexamethyleneimine (HMI) as the OSDA. It is found that the main influencing factor for the transformation of zeolite from rod-like to flake-like morphologies is the ratio of SiO_2_/Al_2_O_3_ at higher crystallization temperature. At the same time, the crystal orientation has changed from *c*-axis orientation to *b*-axis orientation. And the formation of flake-like zeolite is related to the ratio of SiO_2_/Al_2_O_3_, crystallization temperature, silicon source, and structure directing agent. On a macro level, the growth rate of rod-like zeolite is higher than that of flake-like zeolite. And both of them are formed by transforming amorphous precursors into small crystals and growing them epitaxially. On a micro level, as the ratio of SiO_2_/Al_2_O_3_ increases, HMIH^+^ gradually replaces Na^+^ to balance the skeleton charge. DFT calculations and solid-state nuclear magnetic resonance results confirm the specific guidance of HMIH^+^ on the T_2_ and T_4_ sites of rod-like and flake-like mordenites.

## Data availability

The authors confirm that the data supporting the findings of this study are available within the article and ESI.†

## Author contributions

Z. Wang: conceptualization, data curation, methodology, writing – original draft, and writing – review and editing. M. Ke: funding acquisition. Z. Song: supervision. All the authors reviewed the manuscript.

## Conflicts of interest

The authors declare that they have no known competing financial interests or personal relationships that could have influenced the work reported in this paper.

## Supplementary Material

RA-015-D5RA00666J-s001

## References

[cit1] Frilette V. J., Rubin M. K. (1965). J. Catal..

[cit2] Barrer R. M. (1948). J. Am. Chem. Soc..

[cit3] https://www.iza-structure.org/

[cit4] Xiong Z., Qi G., Zhan E., Chu Y., Xu J., Wei J., Ta N., Hao A., Zhou Y., Deng F., Shen W. (2023). Chem.

[cit5] Liu R., Fan B., Zhang W., Wang L., Qi L., Wang Y., Xu S., Yu Z., Wei Y., Liu Z. (2022). Angew. Chem., Int. Ed..

[cit6] Qi X., Chen X., Kong D., Zheng J., Yuan X., Yang D. (2009). Chin. J. Catal..

[cit7] Ma M., Huang X., Zhan E., Zhou Y., Xue H., Shen W. (2017). J. Mater. Chem. A.

[cit8] Li Y., Li Z., Huang S., Cai K., Qu Z., Zhang J., Wang Y., Ma X. (2019). ACS Appl. Mater. Interfaces.

[cit9] Xu L., Sun J. (2016). Adv. Energy Mater..

[cit10] Shao X., Wang S., Zhang X., Li J., Wang N., Wang Z., Yuan Z. (2022). Prog. Chem..

[cit11] Shvets O. V., Konysheva K. M., Shamzhy M. V., Opanasenko M. V., Yaremov P. S., Xiao C., Zou X., Čejka J. (2019). Catal. Today.

[cit12] Lu K., Huang J., Ren L., Li C., Guan Y., Hu B., Xu H., Jiang J., Ma Y., Wu P. (2020). Angew. Chem., Int. Ed..

[cit13] Yuan Y., Wang L., Liu H., Tian P., Yang M., Xu S., Liu Z. (2015). Chin. J. Catal..

[cit14] Kumar M., Berkson Z. J., Clark R. J., Shen Y., Prisco N. A., Zheng Q., Zeng Z., Zheng H., McCusker L. B., Palmer J. C., Chmelka B. F., Rimer J. D. (2019). J. Am. Chem. Soc..

[cit15] Jiang L., Li X., Gong Y., Meng X., Zhang L., Zhai Y., Shang S., Meng L. (2020). Microporous Mesoporous Mater..

[cit16] Liu M., Li Y., Xie Z., Hao Q., Luo Q., Zhang J., Chen H., Dai C., Ma X. (2020). New J. Chem..

[cit17] Zhang H., Zhang H., Wang P., Zhao Y., Shi Z., Zhang Y., Tang Y. (2016). RSC Adv..

[cit18] Narayanan S., Tamizhdurai P., Mangesh V. L., Ragupathi C., Krishnan P. S., Ramesh A. (2021). RSC Adv..

[cit19] Cao S., Shang Y., Liu Y., Wang J., Sun Y., Gong Y., Mo G., Li Z., Liu P. (2021). Microporous Mesoporous Mater..

[cit20] Wei H., Xie S., Liu K., Xin W., Li X., Liu S., Gu S., Liu S., Xu L. (2015). Chin. J. Catal..

[cit21] Zhang S., Cui M., Zhang Y., Mu Y., Lv T., Zheng J., Zhao J., Liu X., Meng C. (2017). Microporous Mesoporous Mater..

[cit22] Jun J. W., Ahmed I., Kim C. U., Jeong K. E., Jeong S. Y., Jhung S. H. (2014). J. Catal..

[cit23] Iorio J. R. D., Li S., Jones C. B., Nimlos C. T., Wang Y., Kunkes E., Vattipalli V., Prasad S., Moini A., Schneider W. F., Gounder R. (2020). J. Am. Chem. Soc..

[cit24] Nimlos C. T., Hoffman A. J., Hur Y. G., Lee B. J., Iorio J. R. D., Hibbitts D. D., Gounder R. (2020). Chem. Mater..

[cit25] Xing B., Ma J., Li R., Jiao H. (2017). Catal. Sci. Technol..

[cit26] Tang X., Chen W., Dong W., Liu Z., Yuan J., Xia H., Yi X., Zheng A. (2022). Catal. Today.

[cit27] Perdew J. P., Burke K., Ernzerhof M. (1996). Phys. Rev. Lett..

[cit28] Delley B. (1990). J. Chem. Phys..

[cit29] Muraoka K., Chaikittisilp W., Yanaba Y., Yoshikawa T., Okubo T. (2018). Angew. Chem..

[cit30] Mayo S. L., Olafson B. D., Goddard W. A. (1990). J. Phys. Chem..

[cit31] Evans A. M., Parent L. R., Flanders N. C., Bisbey R. P., Vitaku E., Kirschner M. S., Schaller R. D., Chen L. X., Gianneschi N. C., Dichtel W. R. (2018). Science.

[cit32] Smith J. D., Scanlan M. M., Chen A. N., Ashberry H. M., Skrabalak S. E. (2020). ACS Nano.

[cit33] Xu H. Q., Wang K., Ding M., Feng D., Jiang H. L., Zhou H. C. (2016). J. Am. Chem. Soc..

[cit34] Jain R., Mallette A. J., Rimer J. D. (2021). J. Am. Chem. Soc..

[cit35] Mintova S., Gilson J. P., Valtchev V. (2013). Nanoscale.

[cit36] Nguyen A. D., Skvara F. (2016). Cem. Concr. Compos..

[cit37] Rozek P., Krol M., Mozgawa W. (2018). Spectrochim. Acta, Part A.

[cit38] Devos J., Shah M. A., Dusselier M. (2021). RSC Adv..

[cit39] Swaddle T. W. (2001). Coord. Chem. Rev..

[cit40] Mlekodaj K., Bernauer M., Olszowka J. E., Klein P., Pashkova V., Dedecek J. (2021). Chem. Mater..

[cit41] Hincapie B. O., Garces L. J., Zhang Q., Sacco A., Suib S. L. (2004). Microporous Mesoporous Mater..

[cit42] Zhang L., Laak A. N. C., Jongh P. E., Jong K. P. (2009). Microporous Mesoporous Mater..

[cit43] Liu W., Wang Y., Bu L., Zhi Y., Wang Z., Yang M., Chu K., Huang Y., Guo N., Qu L., Sang J. (2023). ACS Appl. Nano Mater..

[cit44] Zhang L., Xie S., Xin W., Li X., Liu S., Xu L. (2011). Mater. Res. Bull..

[cit45] Sano T., Wakabayashi S., Oumi Y., Uozumi T. (2001). Microporous Mesoporous Mater..

[cit46] Zhang H., Liu Y., Jiao Z., He M., Wu P. (2009). Ind. Eng. Chem. Res..

[cit47] Li Q., Cong W., Xu C., Zhang S., Wang F., Han D., Wang G., Bing L. (2021). CrystEngComm.

[cit48] Vuono D., Pasqua L., Testa F., Aiello R., Fonseca A., Korányi T. I., Nagy J. B. (2006). Microporous Mesoporous Mater..

[cit49] Liu M. N., Xie Z. X., Luo Q. X., Zhang J., Chen H., Xu L., Sun M., Ma X., Hao Q. Q. (2022). Ind. Eng. Chem. Res..

[cit50] Wang X., Li R., Yu C., Liu Y. (2021). Microporous Mesoporous Mater..

[cit51] Wang J., Cheng X., Guo J., Xu X., Long Y. (2006). Microporous Mesoporous Mater..

[cit52] Li R., Chawla A., Linares N., Sutjianto J. G., Chapman K. W., Martínez J. G., Rimer J. D. (2018). Ind. Eng. Chem. Res..

[cit53] Zhang Q., Li J., Wang X., He G., Li L., Xu J., Mei D., Terasaki O., Yu J. (2023). J. Am. Chem. Soc..

[cit54] Zhao Y., Gu S., Li L., Wang M. (2024). Environ. Pollut..

[cit55] Morsli A., Driole M. F., Cacciaguerra T., Arletti R., Chiche B., Hamidi F., Bengueddach A., Quignard F., Renzo F. D. (2007). Microporous Mesoporous Mater..

[cit56] Wu P., Kan Q. B., Xu N., Wang D. Y., Shang Y. C., Su M. P., Wu T. H. (2003). Acta Chim. Sin..

[cit57] Peng Z., Liu Z., Gao Y., Liu J., Wang D., Liu H., Zhang Y., Li L. (2023). J. Environ. Chem. Eng..

[cit58] Chibani S., Chebbi M., Lebègue S., Bučko T., Badawi M. (2016). J. Chem. Phys..

[cit59] Guo H., Ren J., Feng G., Li C., Peng X., Cao D. (2014). J. Fuel Chem. Technol..

[cit60] Vilhena F. S., Serra R. M., Boix A. V., Ferreira G. B., Carneiro J. W. M. (2016). Comput. Theor. Chem..

[cit61] Lewis D. W., Freeman C. M., Catlow C. R. A. (1995). J. Phys. Chem..

[cit62] Schwalbe-Koda D., Kwon S., Paris C., Bello-Jurado E., Jensen Z., Olivetti E., Willhammar T., Corma A., Román-Leshkov Y., Moliner M., Gómez-Bombarelli R. (2021). Science.

