# Supplementary material for: Exploration on the mechanism of crystal morphology transformation in mordenite
Source: RSC Adv. 2025 Mar 20;15(11):8696–706. doi: 10.1039/d5ra00666j (PMC11924042; doi:10.1039/d5ra00666j)
Supplement: RA-015-D5RA00666J-s001 [file RA-015-D5RA00666J-s001.pdf]

Supplementary Information (SI) for RSC Advances.
This journal is © The Royal Society of Chemistry 2025

Supply information

1 Characterization of crystal seed

Commercial zeolite (SiO_2/Al_2O_3=30) is used as the crystal seed. To be specific, sodium silicate and fume silica are used as silicon sources as well as aluminosilicate is used as aluminum source. And mordenite is dynamically synthesized utilizing HMPA as OSDA. XRD pattern (Figure S1) and SEM image (Figure S2) show that the crystal is the typical nano-mordenite.

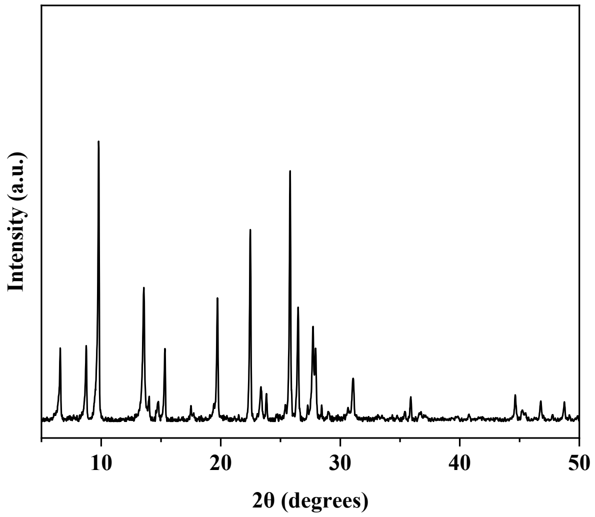

FIGURE S1 XRD parttern of commercial nano-mordenite

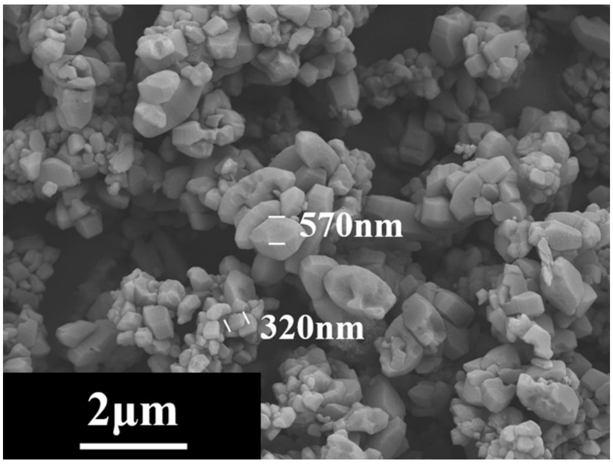

FIGURE S2 SEM image of commercial nano-mordenite

2 Analysis of the growth mechanism of rod-like mordenite

Select crystallization time of 0h, 6h, 12h, 24h and 48h for XRD and SEM analysis. XRD patterns (Figure S3) show a distinct characteristic diffraction of mordenite with a crystallization time of 12 h. The crystallization time of 6h to 12h is the rapid growth period of zeolite, and the growth tends

to be complete at 24h. The SEM image (Figure S4d) also shows that there is basically no amorphous substance present after 24 hours.

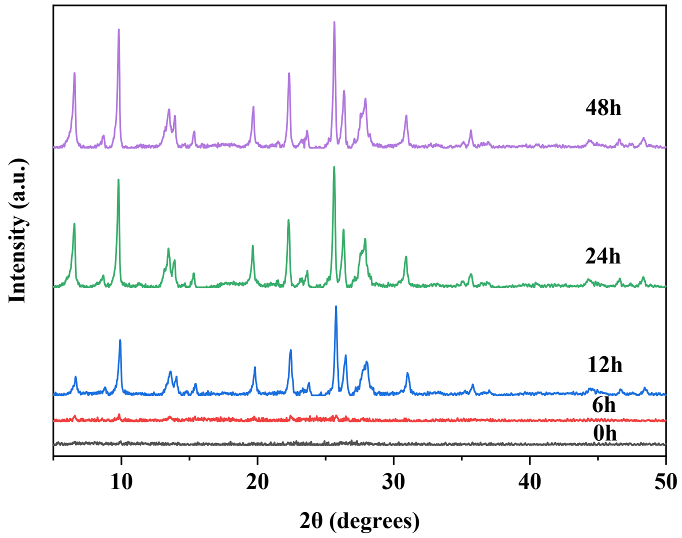

FIGURE S3 XRD partterns of rod-like mordenite with different crystallization time

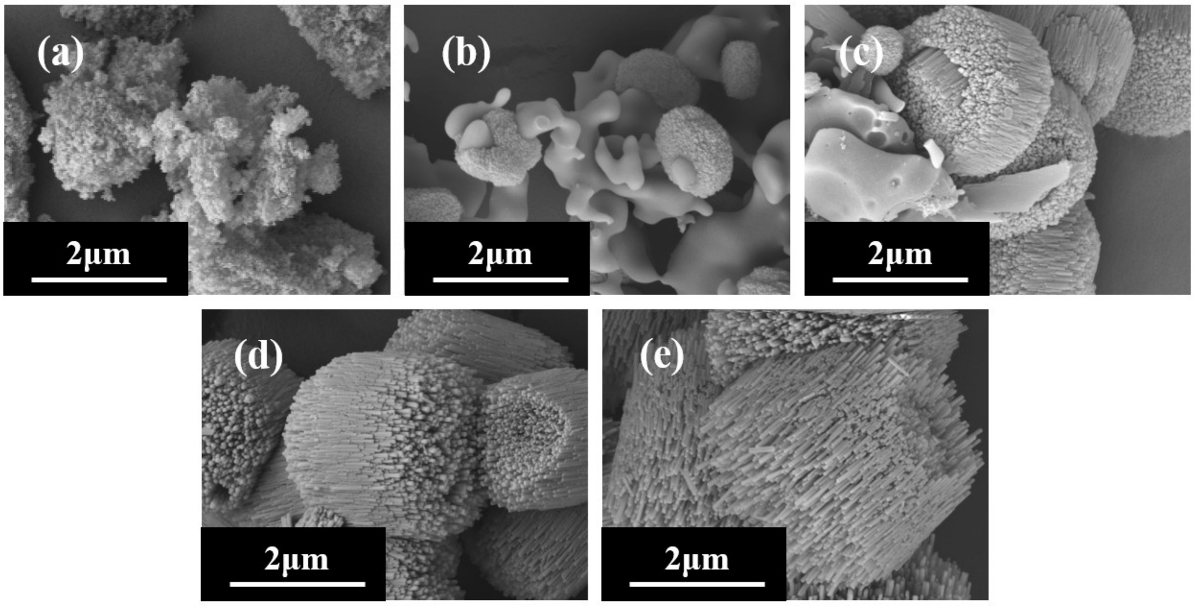

FIGURE S4 SEM images of rod-like mordenite with different crystallization time (a. 0h; b. 6h; c. 12h; d.24h; e.48h)

3 Analysis of the growth mechanism of flake-like mordenite

Select crystallization time of 0h, 12h, 24h, 36h and 48h for XRD and SEM analysis. XRD patterns (Figure S5) show a distinct characteristic diffraction of mordenite with a crystallization time of 24 h. The crystallization time of 24h to 36h is the rapid growth period of zeolite, and the growth tends

to be complete at 36h. The SEM image (Figure S6d) also shows that there is basically no amorphous substance present after 36 hours.

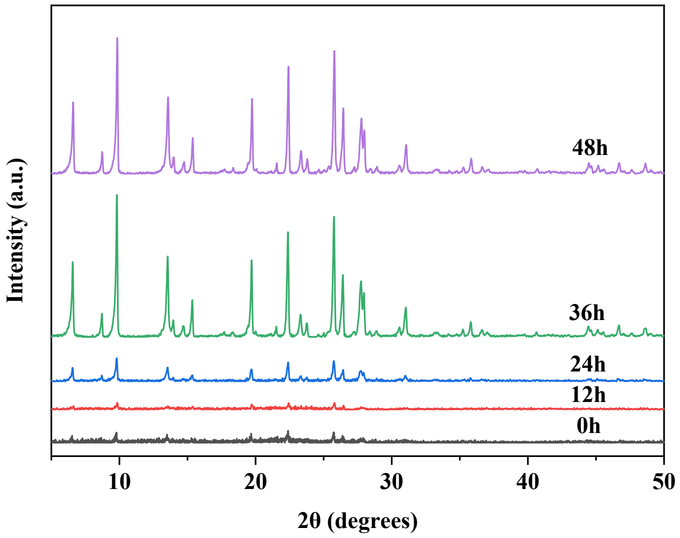

FIGURE S5 XRD partterns of flake-like mordenite with different crystallization time

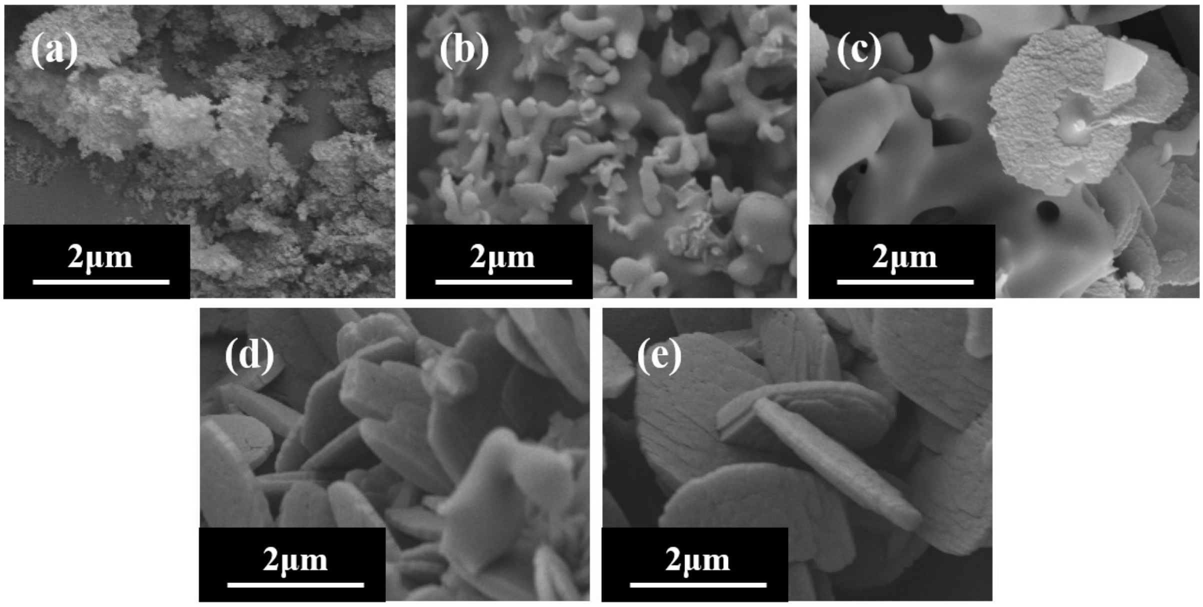

FIGURE S6 SEM images of flake-like mordenite with different crystallization time (a. 0h; b. 12h; c. 24h; d.36h; e.48h)

4 Composition of mordenite with different SiO_2/Al_2O_3 ratios

The compositions of mordenites with different SiO_2/Al_2O_3 ratios synthesized at 170 ℃ are analyzed. Si, Al, and Na elements are analyzed by ICP-OES instrument. And HMI can be estimated by calculating the decrease in weight between 433K and 923K in an air atmosphere [1].

TABLE S1 MOR-x sample composition analysis (mol)

(a.ICP-OES Analysis;b. TG Analysis)

Samples	SiO_2/Al_2O_3[a]	Na/Al[a]	HMI/Al[b]
MOR-14 (170)	6.69	0.95	0.21
MOR-21 (170)	9.14	0.77	0.37
MOR-28 (170)	12.23	0.64	0.49
MOR-35 (170)	13.74	0.61	0.61
MOR-42 (170)	18.77	0.59	0.68

4 Simulation of the different types of mordenite

Compute the substitution energy of H-MOR to compare with Na-MOR and HMI-MOR. The Brönsted centres are chosen as T_1O_7, T_2O_5, T_3O_1, and T_4O_2 respectively [2]. And the initial positions for $HMIH^+$ and Na^+ are determined based on the GCMC simulation result and the literature [3]. The optimized structures are shown in the Figure S7, S8 and S9 respectively.

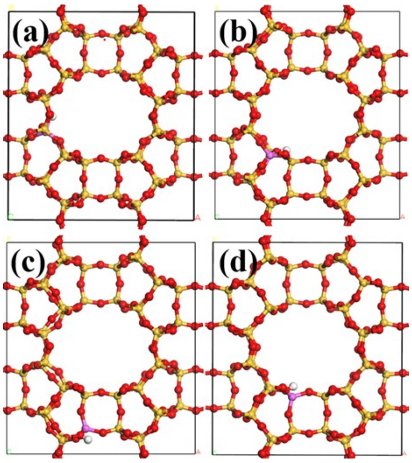

FIGURE S7 Optimized structure of H-type mordenite at different T-sites

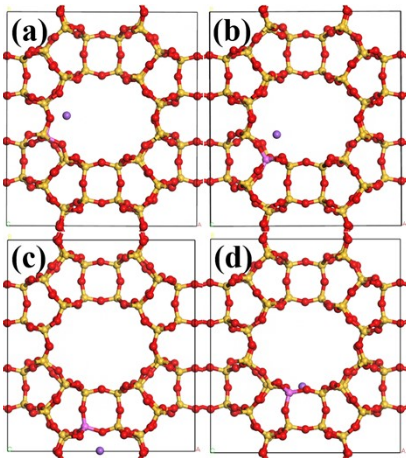

FIGURE S8 Optimized structure of Na-type mordenite at different T-sites

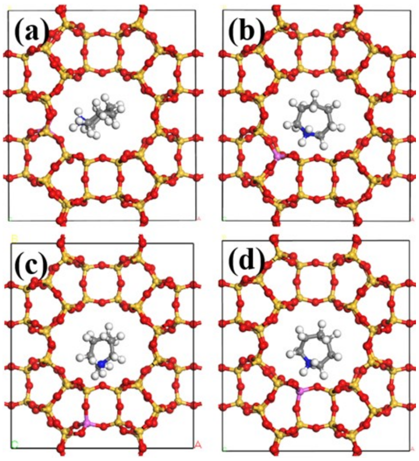

FIGURE S9 Optimized structure of HMI-type mordenite at different T-sites

5 Nuclear magnetic resonance characterization

^{27}Al MAS NMR and 2D ^{27}Al MQ MAS NMR experiments are performed at 18.8 T magnetic fields on spectrometers by Avance neo 600 instrument. The tests are conducted utilizing as-calcined samples. Specifically, ^{27}Al MAS NMR at 25K Hz is performed with 512 sample cycles accompanied by an acquisition time of 0.0434s and a delay time of 2s (Larmor frequencies of 156.4 MHz for ^{27}Al). 2D ^{27}Al MQ MAS NMR experiments are performed at a size of fid of 80 in F_1 and 512 in F_2, and the number of scans is 256. Sampling times of 0.005s for F_1 and 0.05s for F_2 are accompanied by a Dwell time of 10.6μs and a Pre-scan delay of 6.50μs. ^{27}Al MQ MAS and ^{27}Al are acquired at 25K Hz on a 2.5 mm H/X MAS Bruker probe. ^{27}Al and ^{27}Al 3Q MAS use 0.1 M solution of Al $(NO_3)_3$ at 0 ppm as a reference. According to the F_1 dimension information, the chemical shifts of different framework aluminum species in the F_2 dimension are identified as T_3, T_4, T_1 and T_2 respectively [4].

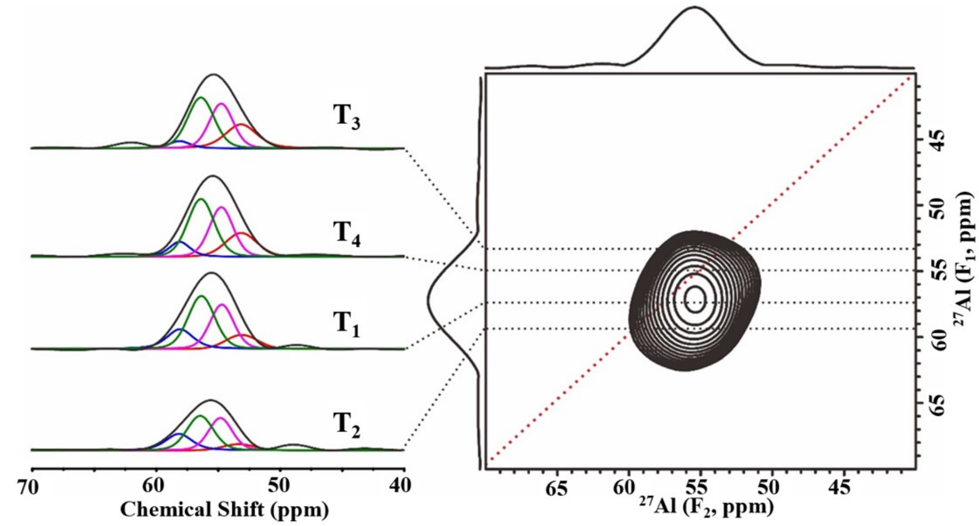

FIGURE S10 ^{27}Al MQMAS NMR spectra of MOR-21(HMI) sample at 18.8 T on the right, and the slices

extracted from F_1 dimension with the fitting lines on the left

TABLE S2 Detailed simulation parameters obtained from the sliced spectra of MQMAS spectra

T site	$F1_{ppm}$[a]	$F2_{ppm}$[b]	σ_{iso}[c]	PQ_{MHz}[d]
T_3	54.0	53.10	53.67	1.52
T_4	55.2	54.70	55.01	1.13
T_1	58.8	56.34	57.89	2.51
T_2	61.7	58.07	60.36	3.05

[a] Chemical shift along F_1 dimension in ppm

[b] Chemical shift along F_2 dimension in ppm

[c] Isotropic chemical shift values obtained by fitting each sliced spectrum

[d] PQ = CQ(1+η2/3)1/2

PQ : Quadrupolar product parameter

The ^{27}Al MAS NMR analyses of samples with different SiO_2/Al_2O_3 ratios with the absence and presence of OSDA are shown in the Figure S11. Based on the analysis results mentioned above, the peak fitting results are shown in the Table S3.

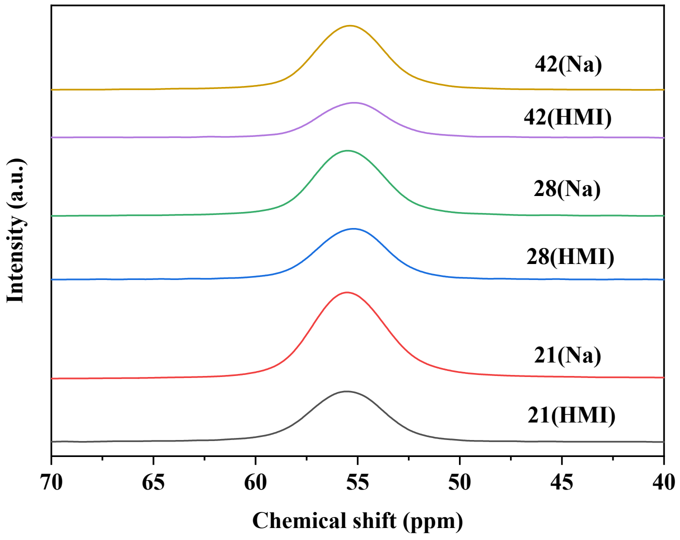

FIGURE S11 ^{27}Al MAS NMR spectra of different samples

TABLE S3 Relative Al content of different samples

SiO_2/Al_2O_3	T_3	T_4	T_1	T_2
**21（HMI）**	15.01	35.30	37.12	12.57

**21（Na）**	18.84	33.81	37.57	9.79
**28（HMI）**	16.46	41.21	34.49	7.84
**28（Na）**	15.79	37.25	38.59	8.36
**42（HMI）**	17.08	41.98	34.61	6.33
**42（Na）**	15.69	39.06	37.31	7.95

6 References

[1] P. Wu, Q. B. Kan, N. Xu, D. Y. Wang, Y. C. Shang, M. P. Su, T. H. Wu, Acta Chimica Sinica, 61(2003) 1202-1207.

[2] H. Guo, J. Ren, G. Feng, C. Li, X. Peng, D. Cao, J. Fuel Chem. Technol., 42(2014) 582-590.

[3] S. Chibani, M. Chebbi, S. Lebègue, T. Bučko, M. Badawi, 144(2016) 244705.

[4] R. Liu, B. Fan, W. Zhang, L. Wang, L. Qi, Y. Wang, S. Xu, Z. Yu*, Y. Wei, Z. Liu, Angew. Chem. Int. Ed., 61(2022) 202116990.
